# The Effect of Xylazine Premedication on the Dose and Quality of Anesthesia Induction with Alfaxalone in Goats

**DOI:** 10.3390/ani11030723

**Published:** 2021-03-06

**Authors:** Mahmoud M. Abouelfetouh, Lingling Liu, Eman Salah, Rui Sun, Sha Nan, Mingxing Ding, Yi Ding

**Affiliations:** 1 College of Veterinary Medicine, Huazhong Agricultural University, No. 1, Shizishan Street, Hongshan District, Wuhan 430070, China; drmahmoud_84@yahoo.com (M.M.A.); liulingling678@163.com (L.L.); sunrui2011111111@outlook.com (R.S.); nansha@webmail.hzau.edu.cn (S.N.); dmx@mail.hzau.edu.cn (M.D.); 2 Department of Surgery, Radiology and Anaesthesiology, Faculty of Veterinary Medicine, Benha University, Moshtohor, Toukh 13736, Egypt; 3 National Reference Laboratory of Veterinary Drug Residues (HZAU) and MAO Key Laboratory for Detection of Veterinary Drug Residues, Huazhong Agricultural University, Wuhan 430070, China; eman.salah@fvtm.bu.edu.eg; 4 Department of Pharmacology, Faculty of Veterinary Medicine, Benha University, Moshtohor, Toukh 13736, Egypt

**Keywords:** alfaxalone, cardiorespiratory parameters, induction, goats, xylazine

## Abstract

**Simple Summary:**

Goats have been used as animal models in research and are increasingly kept as companion animals like dogs and cats. Therefore, keeping them to be always healthy and disease free is an important matter for the owners. Anesthesia is a prerequisite for veterinarians to treat animals suffering from medical and surgical disorders. However, information about the efficacy of anesthetic drugs is scant in this species. In this current study, the quality of induction, antinociceptive and cardio-respiratory effects of a newer anesthetic combination “xylazine–alfaxalone” were evaluated. It is well known that the small opening of the oral cavity and long soft palate make intubation in goats a very challenging procedure. The findings demonstrated that xylazine reduced the dose of alfaxalone required for successful intubation and uneventful induction. In addition, this anesthetic regimen produced better antinociception with stability in cardiac function indices, however oxygen supplementation is necessary, especially in compromised animals, to counteract xylazine-associated lowering of hemoglobin oxygen saturation.

**Abstract:**

Goats have been used as animal models in research and are increasingly kept as companion animals. However, information about effective anesthetic drugs is scarce in this species. The objective of this study was to evaluate the effect of xylazine premedication on alfaxalone induction. Twelve clinically healthy goats weighing 18.5 ± 2 kg were randomly assigned to two groups. Induction was performed with alfaxalone alone intravenously (ALF group) or with xylazine premedication before alfaxalone administration (XYL-ALF group). The quality of induction was scored, induction doses of alfaxalone were determined, and cardiorespiratory parameters and nociceptive thresholds were measured before any treatment(s) (baseline) and at 5, 15, 25 and 35 min after alfaxalone administration. The mean dose of alfaxalone required for induction in the ALF group was greater than that in the XYL-ALF group (*p* < 0.001). There were no significant changes in diastolic arterial pressure (DAP), mean arterial pressure (MAP) or systolic arterial pressure (SAP) compared to baseline in either group, while hemoglobin oxygen saturation (SpO_2_) was lower from 5 to 25 min (*p* < 0.5) in the XYL-ALF group. The nociceptive threshold was significantly higher at 5 min in the XYL-ALF group than in the ALF group (*p* = 0.0417). Xylazine premedication reduced the required dose of alfaxalone for anesthetic induction and produced better antinociception than alfaxalone alone. In addition, the combination of xylazine and alfaxalone allowed for successful induction; however, oxygen supplementation is necessary to counteract xylazine-associated hypoxemia.

## 1. Introduction

Goats have been used as an animal model in many studies, especially in cardiovascular and regeneration, orthopedic and reproductive studies, due to their availability, compliance and relatively low cost. Anesthesia is usually required for further research studies with this species. Using a combination of premedication and general anesthesia is referred to as multimodal anesthesia [1]. The concept of multimodal anesthesia is that the combined drugs may act synergistically to produce anxiolysis, smooth induction and reduce dosage requirements of anesthetic agents, thus minimizing adverse effects of individual drugs. The study of new anesthetic induction regimens can be of interest in goats to provide safer anesthesia with minor complications. The choosing of appropriate anesthetics as well as preanesthetic agents is a pivotal issue in multimodal anesthesia.

Alfaxalone is a neuroactive steroid that is commonly used for anesthetic induction in dogs [2] and cats [3]. It intensifies the inhibitory effects of γ-aminobutyric acid (GABA) on GABA_A_ receptors, causing nerve cell hyperpolarization and blocking neural impulse transmission. Alfaxalone was first sold commercially in combination with alphadolone dissolved in cremophor EL as Saffan for veterinary use. This combination product was later withdrawn from the market because it triggered, in some animals, histamine release that led to hyperemia, oedema and bronchospasm. Recently, a new formulation of alfaxalone, in which alfaxalone is solubilized in 2-hydroxypropyl beta cyclodextrin, has been marketed as Alfaxan. Alfaxalone has several benefits compared to other anesthetic induction agents, including a broad margin of safety and fast onset with good muscle relaxant properties. Furthermore, it does not seem to have less cumulative effects after repeated doses, thereby exhibiting rapid recovery in dogs [4]. Alfaxalone has been also used as both an induction and maintenance agent in other species such as goats [5,6] sheep [7,8], horses [9,10] and donkeys [11]. Alfaxalone anesthesia showed minimal effects on cardiorespiratory variables with prolonged recovery times in sheep and goats [5,12]. Alfaxalone could be also used safely in pregnant ewes with stability in maternal and foetal hemodynamics [13]. Even though alfaxalone has fewer negative impacts on the cardiovascular system, it has a poor analgesic effect and is associated with muscle twitches and seizure-like activity during induction [14,15].

Xylazine is an α_2_ adrenergic receptor agonist that is most commonly used as a sedative agent and an adjunct to general anesthesia in production animal veterinary practice. This drug produces its effects by binding to alpha_2_ adrenergic receptors distributed centrally in the brain or supraspinally (for sedation and some antinociception) and in the dorsal horn of the spinal cord (for antinociception) as well as in the vessel vasculature (for vasoconstriction) [16]. In ruminants, a lower dose of xylazine is needed to produce the same analgesic and sedative effects as in other domestic species, such as horses, donkeys and dogs [17,18]. Xylazine has a very good muscle relaxation effect; however, it can have deleterious effects, notably a negative inotropic effect leading to a decrease in cardiac output (CO) of more than 50% at a therapeutic dose [16].

To our knowledge, there are no scientific reports on the effect of xylazine on the induction dose and physiological effect of alfaxalone in goats. Since xylazine has good muscle relaxation and analgesic effects, we hypothesized that premedication with xylazine would facilitate the intubation procedure, reduce the dose and improve the effect of alfaxalone, improving muscle relaxation and analgesia.

## 2. Material and Methods

### 2.1. Animals

Yichang white goats aged six to eight months and 18.5 ± 2 kg in body weight were submitted to a comprehensive physical examination including a complete blood count, serum biochemistry profile and serological determination of *Brucella melitensis* status. Goats enrolled in this study were acclimatized to being handled and the experimental environment for 2 h daily for 7 days. The goats were fasted for 16–20 h prior to anesthetic induction but allowed free access to water. This study was approved by the animal experimental ethical inspection of laboratory animal center, Huazhong Agricultural University (HZAUGO-2019-001).

### 2.2. Experimental Design

A total of twelve goats were randomly assigned to one of two equal sized groups using a computer program (www.randomizer.org (accessed 25 October 2019) in this prospective, randomized, blinded experiment. In the ALF group, induction was performed with alfaxalone alone intravenously (IV). Goats in the XYL-ALF group were premedicated with xylazine IV (Xylaject 2%, Adwia, Egypt, 0.05 mg/kg bw) 10 min before induction with alfaxalone.

Prior to induction, a 22G 2.5-cm catheter was inserted into each goat’s left cephalic vein. The dose of alfaxalone (Alfaxan, Jurox, Australia, 3 mg/kg bw) [6] with an extra 50% of the dose (1.5 mg/kg bw) was drawn into the syringe. All alfaxalone doses were rounded to one decimal point and injected according to the following protocol: one-quarter (25%) of the calculated dose was administered every 15 s, while a designated person blinded to treatment assessed the jaw and tongue tone. Alfaxalone administration ceased when the jaw tone diminished, and the tongue was flaccid. At that point, endotracheal intubation using silicone with 7.5 mm internal diameter tube was performed in sternal recumbency by the aid of a 30 cm illuminated laryngoscope blade. The endotracheal tube cuff was immediately inflated to an adequate volume that was considered by judging the size of the endotracheal tube relative to the tracheal diameter. The endotracheal tube was removed when the animal resumed a swallowing reaction, then the time of extubation was recorded. The mean amount of alfaxalone required for successful intubation was later recorded. No other drugs were administered to goats during the experiment.

**Figure 1:**
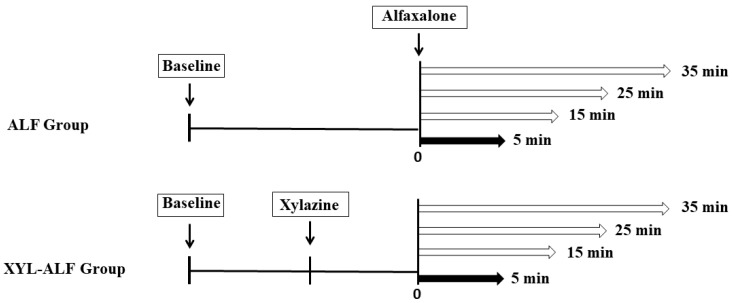
Study timeline illustrating the time of anesthetic induction with IV alfaxalone alone (ALF group) (n = 6) and with alfaxalone following IV xylazine administration (XYL-ALF) (n = 6). The data collection time points were at baseline and at 5, 15, 25 and 35 min after alfaxalone. The pedal withdrawal reflex and end tidal carbon dioxide (P_E_’ CO_2_) were recorded only at 5 min after alfaxalone administration (black arrows).

The induction was scored numerically by an experienced anesthesiologist who was unaware of the drug treatments. The scores were (0) smooth, (1) fair and (2) poor (Appendix A) [19]. The goats were allowed to inhale room air and placed in right lateral recumbency in preparation for echocardiography. All goats were shaved between the 3rd and 6th intercostal spaces on their right thoraxes on the left flank and placed over the cutout in the table. Cardiorespiratory and key echocardiographic parameters, nociceptive threshold of the flank and tone of abdominal musculature were recorded at baseline and at 5, 15, 25 and 35 min after alfaxalone administration (Figure 1). The baseline and experimental values were recorded while the goat was in a recumbent position. Adverse effects during anesthetic induction were also evaluated.

#### 2.2.1. Cardiorespiratory Parameters and Rectal Temperature

Heart rate (HR, beats/min), respiratory rate (*f*_R,_ breaths/min), non-invasive arterial blood pressure (mmHg) and rectal temperature (RT, °C) were recorded using a multiparameter electrocardiogram monitor (PM-9000 Express, Mindary Co., Ltd., Shenzhen, China) with a rectal temperature probe. Systolic (SAP), mean (MAP) and diastolic (DAP) were measured with a 9 cm width cuff wrapped around the thigh of the left pelvic limb close to the inguinal groove to measure the pressure of the femoral artery [20]. A capnography-pulse oximetry monitor (YK-820 MINI-VET, Xuzhou Kangray Electronics Co., Ltd.) was used to monitor hemoglobin oxygen saturation (SpO_2_) with the reflective probe placed over the shaved goat’s tail. Immediately after intubation, the endotracheal tube was connected to a quantitative mainstream CO_2_ sensor (Accuflow^®^) to record end tidal carbon dioxide (P_E_’CO_2_).

#### 2.2.2. Echocardiographic Parameters

Two-dimensional guided M mode echocardiography was performed using an ultrasound machine (Siemens, X-300, Korea). The right parasternal long axis view was used for cardiac imaging. To limit inaccuracies, overestimation and interobserver variability, an experienced cardiac ultrasonographer was responsible for collecting all images. The echocardiographic scanning technique and linear measurement of the left ventricular internal dimensions during diastole (LVIDd) and systole (LVIDs), the interventricular septum dimension (IVSD) and the left ventricular posterior wall dimension (LVPWD) were standardized according to the recommendations from the American Society of Echocardiography and the European Association of Cardiovascular Imaging [21]. The LV internal dimensions were calibrated using ultrasound integrated software, automatically calculating ejection fraction (EF) (%), fractional shortening (FS) (%) and stroke volume (SV) (ml). CO (L/min) was calculated by multiplying the ECG-recorded HR by stroke volume (SV); thus, CO = HR ∗ SV. The cardiac index (L/min/m^2^) was measured by dividing the cardiac output by the animal’s body surface area (BSA). The equation $\mathrm{BSA}=4.688*W^{\left(0.8168-0.0154*\mathrm{logW}\right)}$ was used to estimate BSA, as previously described [22]. (W) refers to the animal’s weight.

#### 2.2.3. Nociceptive Thresholds and Flank Muscle Tone

The nociceptive threshold was measured at the center of the left flank at baseline and 5, 15, 25 and 35 min after alfaxalone administration. In this maneuver, potassium iontophoresis was used to electrically elicit nociception via the gradual influx of potassium ions through the skin. The concentration of ions in subcutaneous tissue was positively proportional to the applied voltage or current [20]. The left flank was shaved, washed with soap and water and degreased with 75% ethanol. Two electrodes connected to the Direct Current Induction Therapy Apparatus (Shantou Medical Equipment Factory Co., Ltd., Shantou, China) were wrapped with gauze tape, soaked in a saturated solution of potassium chloride and applied 1–2 cm apart on the skin. The apparatus was used to deliver pulsed direct current to the electrodes, forcing the entry of potassium ions into subcutaneous tissue. Voltage was increased to the point at which there were obvious contractions of the adjacent skin and muscles and/or the animal attempted to turn its head towards the flank. At that point, the current was discontinued. The pedal withdrawal reflex was scored 5 min after alfaxalone administration using a modified numerical scale (Appendix B) [23]. To assess muscle relaxation, an investigator who was blinded to the administered drugs scored abdominal muscle tone by palpation of the center of the flank (Appendix B) [20]. The scores were 0–4; normal resting tone is represented by a score of 2; less tone was indicated by scores less than 2, while high tone was indicated by scores greater than 2.

### 2.3. Statistical Analysis

Statistical tests were performed using GraphPad Prism software version 5.0. Ordinal data (scores) are presented as the median and range, and continuous data are reported as the mean ± SD. The Kolmogorov–Smirnov test was used to assess the normality (Gaussian distribution) of variables. For normally distributed data (cardiorespiratory, echocardiographic and nociceptive threshold variables), one-way repeated measures ANOVA with Tukey’s post hoc test was used to compare variables within each group, and Student’s *t*-tests were used to compare variables between both groups. Kruskal–Wallis and Mann–Whitney U tests were used to analyze nonparametric data and variables with categorial data (induction, flank muscle tone and pedal nociceptive scores). A *p* value < 0.05 was considered to be statistically significant.

## 3. Results

### 3.1. Sparing Effect of Xylazine on Alfaxalone Induction Dose and Quality

Goats in the ALF group required a mean alfaxalone dose of 4.0 ± 0.30 mg/kg bw for anesthetic induction compared with those in the XYL-ALF group (2.3 ± 0.25 mg/kg bw; *p* < 0.001) (Table 1). During induction, muscle fasciculations and palpebral reflexes were observed in four goats from the ALF group and two goats from the XYL-ALF group. Induction scores in the XYL-ALF group were lower than those in the ALF group, although no statistically significant difference was observed (*p* = 0.3202) (Table 1). Neither ruminal tympany nor regurgitation was found in either group.

### 3.2. Effects of Anesthetic Induction with Alfaxalone Alone or Combined with Xylazine 

#### 3.2.1. Cardio-Respiratory Parameters

The ALF group showed significant variations in HR data at 5 and 15 min compared with the baseline (*p* < 0.001). There was an increase in HR at 15, 25 and 35 min in the ALF group compared to the XYL-ALF group (*p* = 0.0002, 0.0163 and 0.0094, respectively) (Table 2). Following induction, no significant changes were observed in DAP, MAP or SAP over time compared to baseline in either group. Compared with the ALF group, the XYL-ALF group showed decreases in DAP and MAP at 5 and 15 min (*p* = 0.0008 and 0.0184 and 0.0011 and 0.0209, respectively) and in SAP at 5 min (*p* = 0.0085) after alfaxalone administration. P_E_’CO_2_ readings were monitored only at 5 min because it was the minimum time to extubation recorded in the ALF group (6.6 ± 1.9 min) (Table 1). A significant difference in P_E_’CO_2_ (*p* = 0.0049) was found between the two groups.

In the ALF group, there were no significant disparities concerning SpO_2_ during the experiment. However, the SpO_2_ values in the XYL-ALF group at 5, 15 and 25 min were lower (*p* < 0.001 for the first three aforementioned time points and 0.01 for the last time point) than those observed at baseline. The SpO_2_ values were lower at 5, 15 and 25 min (*p* < 0.001, *p* < 0.001 and *p* = 0.0065, respectively) in the XYL-ALF group compared with the ALF group (Table 2). No major differences occurred in *f*_R_ within each group, but considerable dissimilarity (*p* = 0.0124) was noted at 35 min between the two groups. Rectal temperature decreased versus baseline in both groups, but in the XYL-ALF group, the decreases were greater than those observed in the ALF group (*p* < 0.0001 and *p* < 0.001, respectively).

#### 3.2.2. Echocardiographic Parameters

Echocardiographic parameters (EF, FS, SV, CO and CI) were measured to assess left ventricular systolic function. These parameters fluctuated within the clinically acceptable range during the experiment. At 5 min, EF and FS were greater (*p* = 0.0107 and 0.0081, respectively) in the XYL-ALF group than in the ALF group (Table 3).

#### 3.2.3. Nociceptive Thresholds and Flank Muscle Tone 

Nociceptive threshold values in the XYL-ALF group were increased (*p* < 0.05) at 5 min after alfaxalone administration compared with the baseline. Nociceptive thresholds in the ALF group did not significantly change during the experiment. The nociceptive threshold was higher in the XYL-ALF group than in the ALF group and statistically significant (*p* = 0.0417) at 5 min (Table 4). The nociceptive withdrawal reflex in the XYL-ALF group was lower (*p* = 0.0163) than that in the ALF group at 5 min. Flank muscle tone scores in the XYL-ALF group were decreased (*p* < 0.05) at 5 min after alfaxalone administration. Flank muscle tone scores in the ALF group did not significantly change during the experiment. There was a difference (*p* = 0.0198) in the tone of flank muscle between the two groups at 5 min (Table 4).

## 4. Discussion

The alfaxalone dose required for anesthetic induction was approximately 50% lower with the combined use of xylazine and alfaxalone than with alfaxalone alone in the goats. The mean dose of alfaxalone needed to intubate xylazine-sedated goats approached that found in medetomidine-premedicated sheep (2 mg/kg bw) [8], butorphanol-premedicated goats (2.25 mg/kg) [6] and midazolam-premedicated goats (2–2.5 mg/kg bw) [24,25]. This showed that sedative premedication could greatly reduce the dose of drugs required for anesthetic induction.

In the present study, the induction quality score of the XYL-ALF group was lower than that of the ALF group, indicating that more goats that received xylazine and alfaxalone exhibited excitement-free induction and relaxation than goats induced with alfaxalone alone, although there were no statistically significant differences. These observations are consistent with those reported in a prior study [26], while another study reported muscle fasciculations associated with anesthetic induction with alfaxalone in xylazine-sedated horses [10]. Using alfaxalone as a sole induction agent was associated with involuntary muscle contractions [15,27]. In addition, the combined use of high doses (0.015 or 0.03 mg/ kg) of fentanyl and alfaxalone resulted in excitatory behavioral signs during induction [14]. In the current study, the xylazine–alfaxalone combination resulted in the smooth induction of anesthesia in goats. Our induction combination also shows less side effects than other drug combinations reported before. It was found that thiopental or ketamine, when used as induction agents, resulted in severe apnea and regurgitation in goats [28]. However, these adverse signs were not observed in alfaxalone induction with xylazine premedication in our study. 

The increased HR found at 5 min in the ALF group in this trial was similar to that reported in alpacas [15], sheep [7] and dogs [29,30]. In the XYL-ALF group, HR and blood pressure were lower, but still within the normal range at 5 and 15 min, than those in the ALF group. These observations could be attributed to the attenuating effect of xylazine on accelerated HR via central sympatholysis and reduced norepinephrine release from sympathetic nerve terminals [31].

Following induction, no significant changes occurred in SAP, MAP or DAP values in the ALF group. These findings were similar to those observed in previous studies in sheep [6,7]; however, in the XYL-ALF group, SAP and MAP were decreased but remained within the clinically acceptable range. In this study, the stability of arterial blood pressures suggested that increased HR was not a baroreceptor response to hypotension, as previously reported [30,32]. However, it was hypothesized that increased HR acts as a compensatory reflex to maintain CO within the normal range [29,33]. Another study revealed that increased HR was possibly induced via an activating effect of alfaxalone on sympathetic outflow [34]. The *f*_R_ remained unchanged in goats administered alfaxalone alone, but those receiving xylazine and alfaxalone exhibited mild depression in ventilation rate that was considered clinically insignificant [15,35]. In prior reports, anesthesia induction with propofol, ketamine or thiopental revealed a decrease in blood pressure, *f*_R_ and mild increase in HR as well as hypothermia in goats [28,36]. Another report about alfaxalone induction with midazolam and butorphanol premedication showed no significant effect on the SAP, MAP and DAP [6]. Compared with previous reports, the xylazine and alfaxalone combination in our study had less effect on blood pressure and HR than the propofol, ketamine and thiopental combinations and comparable to midazolam, butorphanol and alfaxalone combination. 

In the ALF group, the level of SpO_2_ remained > 90%, similar to that observed in previous studies [27,32]. The XYL-ALF group showed a decrease SpO_2_ with mean values varying from 82% to 87% for 25 min post-induction; however, no significant clinical signs of cyanosis were observed. The absence of cyanosis might be due to α_2_ mediated peripheral vasoconstriction [37]. PE’CO_2_ values at 5 min were higher in the XYL-ALF group compared with the ALF group, but they remained within the normal reference range >20 mmHg and <60 mmHg. In this study, a new anesthetic combination was used, so we decided to not provide supplemental oxygen to the goats to evaluate the potential effect of this combination on this parameter [23]. Anesthesia induced with a lidocaine–alfaxalone combination revealed a degree of lowered arterial oxygen saturation (SaO_2_) ranging from 63.4% to 93.6% at 2 min post-induction [38]. Medetomidine-azaperone-alfaxalone decreased the level of oxygenation with SaO_2_ (66%–88%) for 40 min in white-tailed deer [39]. RT decreased in both groups; however, in the XYL-ALF group, an unexpectedly higher value was observed at 5 min after alfaxalone administration compared with the baseline. Although the difference (0.6) was clinically negligible, it could be explained by the xylazine–alfaxalone combination synergistically preventing core heat loss via peripheral vasoconstriction. 

Echocardiographic parameters showed reductions in EF and FS reported at 5 min in the ALF group, which could be attributed to accelerated HR that, in sequence, did not allow the left ventricle to be filled freely during diastole and decreased the ventricular internal dimensions. Furthermore, it was assumed that CO is likely to be maintained or minimally increased after alfaxalone administration due to a transient increase in HR and increased venous return along with reduced venous compliance [33,34]. A recent study revealed a significant decrease in HR, FS and CO post xylazine sedation [40]. In this study, the echocardiographic parameters did not show any significant change in the XYL-ALF group, indicating that premedication with xylazine before alfaxalone induction has less impact on the cardiovascular system than ALF induction alone. 

In the present study, the potassium iontophoresis method was chosen to objectively record the nociceptive threshold in goats. Potassium iontophoresis provides a reliable non-invasive tool for inducing ideal pain stimuli, which can be presented rapidly and repeatedly over a short period of time with a minimal carry-over effect with repeated trials [41]. In the ALF group, an insignificant increase in nociceptive threshold was noted at 5 min. By comparison, the XYL-ALF group showed a higher nociceptive threshold with noteworthy values at 5 min after alfaxalone administration. These findings confirmed that the xylazine combination with alfaxalone provides synergistic antinociceptive effects in goats. Nociceptive threshold testing revealed that xylazine produces satisfactory antinociceptive effects [42], and alfaxalone has a minimal analgesic effect. This supports that alfaxalone alone is not enough to induce excitement-free induction and abolish nociception of surgical stimuli. It must be combined with a potent analgesic agent to provide additional coverage. Taken together, the combination of xylazine and alfaxalone could achieve smooth induction with satisfactory multimodal antinociception. However, oxygen supplementation was recommended to counterpoise xylazine-associated hypoxemia in goats. 

The present study had several limitations. Firstly, the small sample size may have limited the value of observations as a true representation of the goat population. Secondly, an invasive arterial blood pressure monitoring, and arterial blood gas analysis may better evaluate the cardiopulmonary status. Thirdly, electrical stimulation used to measure the nociceptive threshold may excite all nerve endings, not just those associated with pain stimulation, and could be a confounding factor in evaluating pain threshold. However, this article could provide valuable information about the pros and cons of xylazine and alfaxalone combination in goat anesthetic induction. 

## 5. Conclusions

This research focused on evaluating anesthetic induction with alfaxalone in xylazine-premedicated goats. Administration of alfaxalone following xylazine premedication reduced the induction dose and provided satisfactory induction with better antinociception and muscle relaxation. The combination also has less impact on cardiovascular function. However, blood oxygen tension was clinically decreased in the xylazine premedication group; therefore, oxygen therapy was recommended to preclude xylazine-associated hypoxemia.

## Figures and Tables

**Table 1:** Dose requirements and quality of anesthetic induction with IV alfaxalone alone (ALF group) or with xylazine (0.05 mg/kg bw; IV) and alfaxalone (XYL-ALF group) in goats.

	ALF Group (*n* = 6)	XYL-ALF Group (*n* = 6)	*p* Value
Induction dose (mg/kg bw)	4.0 ± 0.30	2.3 ± 0.25 *	*p* < 0.001
Induction score	1(0–1)	0.5(0–1)	*p* = 0.3202
Time to extubation (min)	6.6 ±1.9	9.4 ± 3.2	*p* = 0.0781

Data are expressed as the median and range (scores), mean ± SD (doses and times). * Significant difference between the two groups (*p* < 0.05).

**Table 2:** Cardiorespiratory variables and rectal temperature measured in goats at baseline and at 5, 15, 25 and 35 min after alfaxalone administration in the ALF group (4.0 ± 0.30 mg/kg bw; IV) (n = 6) and the XYL-ALF group (2.3 ± 0.25 mg/kg bw; IV) (n = 6).

Parameter	Group	Baseline	(5)	(15)	(25)	(35)
HR (beats/min)	ALF	103 ± 10	140 ± 17 *	138 ± 16 *	124 ± 13.3	117 ± 14.4
	XYL-ALF	97 ± 17.2	118 ± 26 *	98 ± 10.2 ^†^	98 ± 23 ^†^	94 ± 14 ^†^
SAP (mmHg)	ALF XYL-ALF	114 ± 10 115 ± 8	124 ± 7 99 ± 15 ^†^ *p* = 0.0085	108 ± 12 101 ± 13	111 ± 8 106 ± 10	113 ± 17 102 ± 9
MAP (mm Hg)	ALF	87 ± 7.9	93 ± 6	83 ± 9.7	85 ± 14	79 ± 18
	XYL-ALF	82 ± 10	68 ± 13.5 ^†^	70 ± 8 ^†^	76 ± 13	73 ± 9.6
DAP (mmHg)	ALF XYL-ALF	70 ± 12 62 ± 8	76 ± 8 53 ± 11 ^†^ *p* = 0.0011	66 ± 9 55 ± 9 ^†^ *p* = 0.0209	72 ± 16 60 ± 18	62 ± 15 67 ± 9
DAP (mmHg)	ALF XYL-ALF	70 ± 12 62 ± 8	76 ± 8 53 ± 11 ^†^ *p* = 0.0011	66 ± 9 55 ± 9 ^†^ *p* = 0.0209	72 ± 16 60 ± 18	62 ± 15 67 ± 9
*f*_R_ (breaths/min)	ALF	20 ± 2.9	22 ± 3.8	21 ± 4.5	21 ± 9.8	22 ± 6.6
	XYL-ALF	23 ± 6.8	16 ± 6	18 ± 4.3	19 ± 8.5	16 ± 2.4 ^†^
SpO_2_ (%)	ALF	95 ± 2.4	90 ± 1.7	92 ± 1.9	93 ± 3	92 ± 3.4
	XYL-ALF	94 ± 2.3	82 ± 1.7 *^†^	84 ± 2.7 ^†^*	87 ± 3 ^†^*	91 ± 2
P_E_’CO_2_ (mmHg)	ALF XYL-ALF		35 ± 1.9 40 ± 2.9 ^†^			
RT (°C)	ALF	39.4 ± 0.3	39 ± 0.5 *	39 ± 0.4 *	39.1 ± 0.4	39.2 ± 0.4
	XYL-ALF	39.4 ± 0.2	40 ± 0.2 *	38.5 ± 0.3 *	38.4 ± 0.3 *	38.4 ± 0.2 *

Heart rate (HR), systolic arterial blood pressure (SAP), mean arterial pressure (MAP), diastolic arterial blood pressure (DAP), respiratory rate (*f*_R_), hemoglobin oxygen saturation (SpO_2_), end tidal carbon dioxide (PE’CO_2_) and rectal temperature (RT). Data are expressed as the mean ± SD. ^†^ Significant difference between the two groups (*p* < 0.5). * Significant difference compared to baseline within the group (*p* < 0.5).

**Table 3:** Echocardiographic variables measured in goats at baseline and at 5, 15, 25 and 35 min after alfaxalone administration in the ALF group (4.0 ± 0.30 mg/kg bw; IV) (n = 6) and the XYL-ALF group (2.3 ± 0.25 mg/kg bw; IV) (n = 6).

Parameter	Group	Baseline	(5)	(15)	(25)	(35)
EF (%)	ALF	76 ± 6	71 ± 8	73 ± 9	75 ± 7	76 ± 8
	XYL-ALF	80 ± 6	84 ± 9 ^†^	78 ± 6	72 ± 9	81 ± 11
FS (%)	ALF	38 ± 8	34 ± 6	36 ± 7	37 ± 6	39 ± 7
	XYL-ALF	42 ± 7	47 ± 9 ^†^	39 ± 6	36 ± 7	44 ± 9
SV (mL)	ALF	18 ± 5	17 ± 9	12 ± 9	16 ± 8	16 ± 3
	XYL-ALF	19 ± 4	14 ± 7	14 ± 8	14 ± 2	18 ± 8
CO (L/min)	ALF	1.8 ± 0.5	2.4 ± 1.3	1.8 ±1.4	2 ± 0.9	2 ± 0.9
	XYL-ALF	1.7 ± 0.2	1.6 ± 0.6	1.3 ± 0.7	1.4 ± 0.4	1.4 ± 0.5
CI (L/min/m^2^)	ALF	2.4 ± 0.6	3 ± 1.5	2.3 ± 1.7	2.5 ± 1	2.5 ± 0.4
	XYL-ALF	2.4 ± 0.3	2.3 ± 0.9	1.8 ± 1	1.9 ± 0.4	1.9 ± 0.8

Ejection fraction (EF), fraction shortening (FS), stroke volume (SV), cardiac output (CO) and cardiac index (CI). Data are expressed as the mean ± SD.^†^ Significant difference between the two groups (*p* < 0.5).

**Table 4:** Nociceptive threshold variables and flank muscle tone scores measured in goats at baseline and at 5, 15, 25 and 35 min after alfaxalone administration in the ALF group (4.0 ± 0.30 mg/kg bw; IV) (n = 6) and the XYL-ALF group (2.3 ± 0.25 mg/kg bw; IV) (n = 6).

Parameter	Group	Baseline	(5)	(15)	(25)	(35)
Nociceptive threshold	ALF	33 ± 14	43 ± 23	36 ± 21	28 ± 5	28 ± 3
	XYL-ALF	29 ± 7	87 ± 52 *^†^	45 ± 28	38 ± 21	35 ± 11
Flank muscle tone	ALF XYL-ALF	2(2) 2(2)	1 (1–2) 0.5 (0–1) *^†^ ^†^ *p* = 0.0198	2 (2–3) 1(0–2) ^†^ ^†^ *p* = 0.0104	2 (2–3) 2 (1–2) *p* = 0.2184	2 (2–3) 2 (2) *p* = 0.5000
Nociceptive response	ALF XYL-ALF		2(1–2) 1(0–2) ^†^ ^†^ *p* = 0.0163			

Data are expressed as the mean ± SD. * Significant difference compared to baseline within the group (*p* < 0.5). ^†^ significant difference between the two groups (*p* < 0.5).

## Data Availability

The data set used for statistical analysis is available upon reasonable request.

## Appendix A

**Table A1:** Induction quality scoring system.

Score	Description
Smooth (0)	No signs of excitement; no palpebral blinking; may or may not be associated with minimal jaw movement during manipulation; clear laryngeal structure visualization and complete tongue flaccidity with easy intubation performed within ten seconds.
Fair (1)	Slight excitement; some rapid blinking, some limb movements and obvious jaw movement during manipulation; intubation is performed within 30–60 s with or without some jaw tone persistence.
Poor (2)	Marked excitement; muscle twitching; paddling of limbs; unable to place orotracheal tube.

Modified from Dzikiti et al., 2014.

## Appendix B

**Table A2:** Pedal withdrawal response and flank muscle tone rating systems.

Rating System	Score: Description
Pedal withdrawal reflex ^1^	(3) Exaggerated response: Active withdrawal of stimulated leg with active movement of head. (2) Weak response: Slow withdrawal of stimulated leg with sluggish head movement. (1) Very weak response: Slow withdrawal of stimulated leg alone. (0) No response: Inactive limb and head reflex
Flank muscle tone ^2^	(0): Complete relaxation to palpation with minimal or no tone palpable. (1): Detectable relaxation to palpation with less tone palpable. (2): Normal resting tone: flank has some elasticity to palpation. (3): Mild resistance and tone with gentle pressure on the skin and muscle. (4): Strong resistance and tone with strong pressure on the skin.

^1^ Modified from Rodrigo-Mocholí et al., 2016; ^2^ Cui et al., 2017.
